# A Fatal Case of Noma in an Adult

**Published:** 1893-01

**Authors:** 


A FATAL CASE OF NOMA IN AN ADULT.—Hansson (*Centralbl. f. Chirurgie*, 1892, No. 41, p 845) has reported the case of a man, fifty-five years old, who had the carious first molar tooth of the upper jaw removed. There was also a pretty firm œdematous swelling of the entire right cheek, and inspection of the mouth disclosed the existence of a stomatitis. Energetic antiphlogistic measures were at once instituted, but the swelling of the cheek progressed in intensity, the lower eyelid becoming involved. The skin of the cheek was tense, pale, and of a wary appearance. In the mouth, opposite the site of the extracted tooth, was a necrotic area as large as a cherry stone. This was excised, and a thorough application of silver nitrate made. The mouth was frequently washed with antiseptic solutions. The swelling diminished somewhat, but the gangrene extended. The discharge of saliva was abundant and offensive. The gangrenous tissue was again excised, but the process was not controlled; on the contrary, it extended to the neck, to the nose, and to the ear. The gangrene became more profound; symptoms of septicæmia appeared; and a purulent bronchitis developed to which the patient succumbed. An autopsy could not be held.

